# Dynamics and vibrational spectroscopy of quasi-one dimensional water wires inside carbon nanotubes of different diameter and chirality

**DOI:** 10.1038/s41598-025-14266-8

**Published:** 2025-08-01

**Authors:** Deepak Ojha, Peter Saalfrank

**Affiliations:** Institute of Chemistry, Potsdam University, Karl-Liebknecht-Strasse 24-25, D-14476 Potsdam-Golm, Germany

**Keywords:** Computational chemistry, Density functional theory, Molecular dynamics, Statistical mechanics

## Abstract

Water strongly confined in nanostructures such as carbon nanotubes (CNTs) exhibits structural, dielectric, transport, dynamical and thermodynamical properties vastly different from bulk water, due to a strong modification of the (three-dimensional) hydrogen bond network. In this work, we mainly address the following aspects of extremely confined, quasi-one dimensional water chains in CNTs which have have not been emphasized much so far: The effect of chirality of the CNT, strong interactions with the hydrophobic walls and the (altered) vibrational response of confined water. Specifically, we have studied the (i) translation / diffusion, (ii) rotation / reorientation and (iii) vibrations of water chains confined within narrow carbon nanotubes (CNTs) with chirality indices (6,2), (6,4) and (6,6) using ab initio molecular dynamics. Special emphasis is on vibrational spectra, notably in the OH stretch region, obtained from fluctuations in the local OH stretching modes which were further employed to obtain two-dimensional infrared spectra and frequency-frequency correlation functions. We find that the vibrational distribution of water molecules under confinement is overall blue-shifted in comparison to bulk water, due to a breakdown of the three-dimensional hydrogen bond network. Further, the vibrational dynamics were found to dependent strongly upon the chirality and diameter of the CNTs, the latter causing stronger hydrophobic interactions with the walls of the nanotube. With respect to translational and rotational motion, the CNT-confined water molecules exhibit slower translational diffusion and faster reorientational motion compared to bulk liquid water for all cases simulated in this work.

## Introduction

Water confined in hydrophobic, carbonic nanostructures such as carbon nanotubes (CNTs) or between graphene sheets has attracted considerable scientific interest in the past, both from theory and experiment. This is due to many (potential) applications on the one-hand side, ranging from nanofluidics over desalination to geology, medicine and biology. On the other-hand side interest is triggered because confined water can show vastly different structural, transport, friction, dielectric, dynamical, spectroscopic and phase transition behaviour compared to bulk water. In Refs.^1–3^ recent reviews specifically for molecular dynamics work on water in CNTs can be found, which is also the focus of this work.

For example, water in not too narrow CNTs shows faster diffusion rates in comparison to bulk water, notably along the nanotube axis^4–15^. For CNTs, on the other hand, with chirality index (9,9) water molecules confined within exhibited reduced diffusion rates even smaller than for the bulk (see below and Ref.^16^ and references therein). Hummer and coworkers in early classical simulation studies on quasi-one dimensional water wires in narrow, open CNTs, demonstrated a “pulse-like” transmission of tightly bound packages (“files”) of water molecules through them^4,5^. Netz and coworkers observed for water-filled CNTs of various diameters not only large effects on diffusion and friction coefficients, but on other properties as well: With decreasing diameter of (and water load inside) CNTs showed decreasing friction coefficients and an increasing giant axial dielectric constant while the radial dielectric constant decreased^17^. In experiments, translational diffusion of water confined within the CNTs is generally measured using pulse-field gradient nuclear magnetic resonance (PFG-NMR) spectroscopy^18^. The PFG-NMR of water confined within double-walled CNT at 263 K reported the self diffusion coefficient to be 4.1 ± 0.2 $\times$ 10$^{-10}$ $\mathrm {m^{2}s^{-1}}$ which is comparable to diffusion of supercooled liquid water at the same temperature with a value of 7.0 $\times$ 10$^{-10} \mathrm {m^{2}s^{-1}}$^18^. Confinement of water close to hydrophobic surfaces, such as those of CNTs or graphene, can lead to stabilization of a liquid phase even at extreme pressure conditions^7^. On the other hand, simulations have reported that quasi 2D-phases of ice not existing under ambient conditions can be generated under confinement^8^.

From an experimental perspective, a quasi elastic neutron scattering (QENS) study of water confined within the CNTs or large diameter demonstrated that at low temperatures the rotational energy of water molecules was identical to that of gaseous water molecule owing to reduced hydrogen bonding along the axis of CNT^19^. In Ref.^3^, MD revealed that orientational relaxation of water molecules arranged in 1D “files” in thin, open ((6,6)) CNTs of various lengths, measured by collective dipolar correlations, was ultraslow at an order of several ns. Similarly, experimental dielectric relaxation measurement of water confined within the CNTs of length ranging in between 0.5 to 3 $\mu m$ and diameter of 1.4 nm reported higher Kirkwood correlation factor on account of higher dipolar correlation in between quasi one-dimensional water wires^20^. The agreement between the experiments and MD simulations strongly depend on the observable being measured in experiment and simulations. Simulations are able to explain and likewise predict the trends in experimental studies of structure and dynamics of water filled CNTs. Nevertheless, there may exist sizable quantitative disagreement between the experiments and their MD benchmarks

Several of observations discussed ahead have been associated with a drastically changed hydrogen bond (HB) network in nano-confined water compared to bulk liquid water. Under strong confinement, water does not form extended three-dimensional hydrogen bond networks but strongly anisotropic networks instead. This is also the case for the water-filled CNTs to be studied in this work (see below).

The strong anisotropy in the HB network significantly influences the translational, vibrational and reorientational motion of water^3,11,12^, and of associated properties. As a consequence of altered vibrational dynamics, also vibrational spectra of confined water molecules are affected. For example, vibrational spectra of water confined within CNTs were found to show stronger intensity in a blue-shifted, higher-frequency domain of 3600-3900 $\hbox {cm}^{-1}$, confirming the presence of “free” or dangling hydrogen bonds^21–23^ which are absent in bulk water. This is also supported by simulations which have demonstrated a reduction in the number of hydrogen bonds (which shift OH frequencies to the red), between confined water molecules^22,23^.

Many of the calculations mentioned above are based on classical molecular dynamics (MD), employing empirical forcefields. Their transferability and applicability to extreme confinement is largely dependent upon the validity of the forcefield employed. Further, carbon nanotubes of different chirality demonstrate metallic (for “armchair” CNTs with chirality indices (n,n)), or semi-conducting behaviour (for most chiral CNTs with chirality index (n,m) and n$\ne$ m)^24–26^. Since change in their chirality indices leads to change in the band structure of CNTs, classical models might not be suitable to study the CNT-water dynamics in CNTs of different chirality. In the present work, density-functional theory based molecular dynamics (ab-initio MD, AIMD) will be used instead which directly incorporates the electronic wavefunction of the system during “on-the-fly” dynamics.

**Fig. 1:**
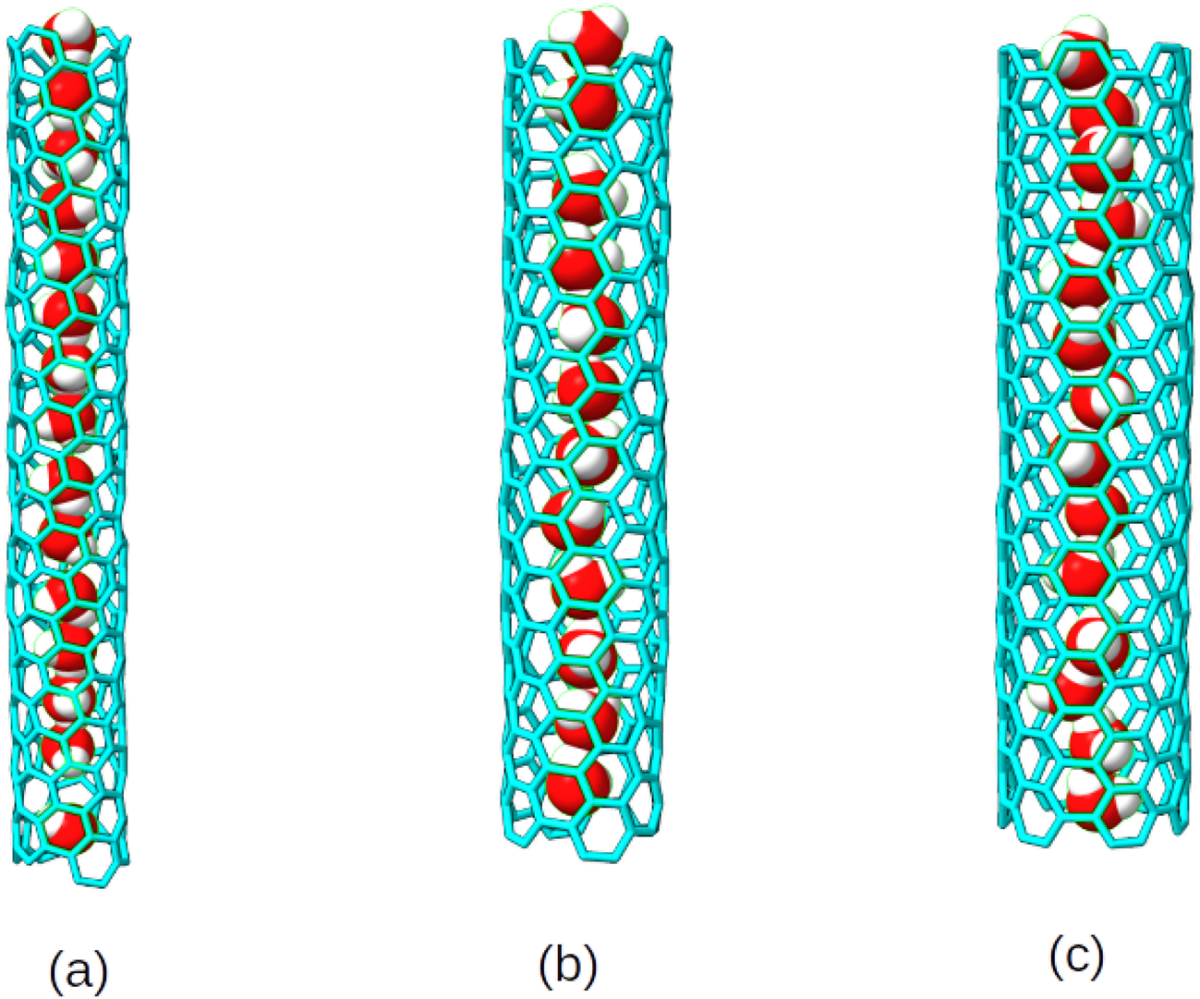
Snapshots of water molecules confined within CNTs of chirality (**a**) (6,2), (**b**) (6,4) and (**c**) (6,6) obtained from AIMD / BLYP+D3 / NVT (T=300 K) simulations.

Our focus is on the translational, rotational and vibrational properties of water within CNTs with special emphasis on the chirality of the CNTs, and on interactions of water with the hydrophobic CNT walls. To this end, one-dimensional water wires confined within narrow carbon nanotubes with chirality indices (6,2), (6,4) and (6,6) will be considered. (6,2) and (6,4) are chiral while (6,6) is of the “armchair” type. The three CNTs have small diameters between about six to nine Å which can accommodate quasi-one dimensional water wires only, similar to those studied by Hummer in Ref.^4^. Due to an increasing diameter of CNTs from (6,2) to (6,6), the average distances between water molecules and the hydrophobic CNT walls increase also, which again affects dynamics. Representative snapshots of the model systems used are shown in Fig.1 below.

While translational motion along the CNT axis is analyzed using diffusion coefficients obtained from mean-square displacements, reorientational dynamics of water molecules is obtained using correlation functions of the second-order Legendre polynomial of the dipole vector of water molecules which can experimentally be obtained by nuclear magnetic resonance spectroscopy^15^. Vibrational frequencies of the OH modes are obtained by performing short-window wavelet transforms of time series generated from the simulation trajectories. Further, two-dimensional infrared (2D IR) spectra were obtained. The timescale at which correlation within intramolecular OH modes is lost is determined from frequency correlation functions, 2D IR spectra and the slope of vibrational echo intensities.

Our paper is organized as follows. In Sec.Computational details the AIMD protocol is described along with the methods to compute the vibrational response of water chains in narrow CNTs with different chirality. Sec.Results and discussion presents results, for dynamics (resulting in diffusion and orientational redistribution) and vibrational spectroscopy, time-averaged and time-resolved. A final section Summary and conclusions summarizes and concludes this work.

## Computational details

### AIMD simulations

Simulation of water ($\mathrm {H_{2}O}$) confined in narrow carbon nanotubes corresponding to chirality indices (6,2), (6,4) (both chiral) and (6,6) (armchair) were performed using the CP2K program package^27^. Supercell geometries were used with large vacuum gaps between CNTs, and periodic boundary conditions along the direction of CNT axes. Representative snapshots (obtained with the methods described below) are shown in Fig. 1. As already said, corresponding to the small diameters of the considered CNTs, ranging from 6.2 to 8.7 Å, only one-dimensional water chains can be accommodated inside the CNTs. The number of water molecules forming the water wires, the diameters of the CNTs, and the dimensions of simulation boxes are given in Table 1. Closer inspection of Fig. 1 shows, that of the two hydrogen atoms of each $\hbox {H}_2$O molecule in the molecular chains in average one is H-bonded to a neighbour molecule, while the other one remains “free”^3^, interacting more or less strongly (depending on the CNT’s diameter) with the hydrophobic CNT walls. Thus the 3D HB network of bulk water is replaced by a quasi-1D, strongly anisotropic HB network. The number of water molecules confined within each CNT was determined based on the overall stability of the linear one-dimensional water wires under CNT confinement using classical forcefield based optimization. Further it was also ensured that the resulting one-dimensional water wire remained continuous throughout the course of AIMD simulation, without splitting into two segments separated by a void.

**Table 1:** The dimensions of simulation boxes, diameters of CNTs, and the number of water molecules forming the water wires for the three CNTs considered in this work, with chirality index (m,n).

(m,n) index	Number of $\mathrm {H_{2}O}$	Box dimensions (Å)	Diameter (Å)
(6,2)	15	20.0 $\times$ 20.0 $\times$ 46.01	6.21
(6,4)	12	20.0 $\times$ 20.0 $\times$ 37.01	7.25
(6,6)	14	20.0 $\times$ 20.0 $\times$ 36.84	8.65

The initial configuration of each system was equilibrated in the NVT ensemble using the CSVR (Canonical Sampling using Velocity Rescaling) thermostat^28^ at a temperature of 300 K for a duration of 10 ps (timestep 0.5 fs) using AIMD. The electronic structure calculations were done using the QUICKSTEP module which is based on a mixed Gaussian and plane waves (GPW) approach^29^. While the Kohn-Sham orbitals are expanded in terms of contracted Gaussians using the DZVP-MOLOPT basis set^30^, the electron density is represented using plane waves, with an energy cutoff of 300 Ry. The effect of nuclei and core electrons was taken into account using norm-conserving Goedecker-Teter-Hutter (GTH) pseudopotentials^31,32^. The BLYP functional was employed for the exchange-correlation functional^33,34^, and Grimme’s D3 correction for dispersion corrections^35^. Earlier AIMD simulations of water have shown that the structure, dynamics, phase diagram, vibrational spectroscopy, hydrogen-bond lifetime and self diffusion is accurately predicted with on incorporation of D3 dispersion corrections bringing it in close agreement with experimentally known results^36^. Finally, in a “production phase” the three equilibrated systems in Table 1 were further simulated in an NVE ensemble for 50 ps each, using a timestep of 0.5 femtoseconds. Thus, for CNTs with at least 12 water molecules per cell (see Table 1) and considering that each water molecule has two OH modes, we have at least 24 trajectories of OH mode of 50 ps duration each accounting in total $50 \times 2 \times 12 =1200$ ps of OH modes simulation trajectory for statistically sampling the dynamics of OH modes of water molecules confined within the CNTs – the main focus in this work. This covers well the time constants associated with the reorientational and vibrational motion of OH modes of a few picoseconds (see below). For comparative analysis, $\mathrm {H_{2}O}$ corresponding to liquid water density at 300 K was also simulated, using 125 water molecules in a cubic box with box size of 15.54 Å, and otherwise the same protocol as for the water-filled CNTs.

### Calculation of properties

From the “production phase”, besides information on OH vibrations (see below), also other properties were calculated.

As a measure for *translational* dynamics, diffusion coefficients (${D_{z}}$) of water molecules along the *z*-axis (main axis of the CNTs) were obtained from the slope of atomic mean square displacements (MSD), as

1 $$\begin{aligned} D_z = \displaystyle \lim _{ t \rightarrow \infty } \frac{1}{2 t} \ \sum _{i=1}^{N} \left| \langle r_{z,i}(t)-r_{z,i}(0) \rangle \right| ^2\end{aligned}$$

Here, ${r_{z,i}(t)}$ is the *z*-coordinate of position vector $\underline{r}_i$ of atom *i* at time *t*, *N* is the number of atoms within the one-dimensional water wire, and the factor 1/2 accounts for 1D diffusion.

Further we quantified the *rotational* / orientational dynamics of water molecules along the CNT axis using orientational correlation functions, defined as^37^,

2 $$\begin{aligned} C_{2}(t) = \frac{\left\langle P_{2}\left( \underline{e}(t) \underline{e}(0) \right) \right\rangle }{\left\langle P_{2}\left( \underline{e}(0) \underline{e}(0) \right) \right\rangle },\end{aligned}$$

where $P_{2}(x)=1/2 (3x^2-1)$ is the Legendre polynomial of rank 2 and $\underline{e}(t)$ the dipole vector of water molecules at time *t*. The $\langle \cdots \rangle$ indicates averaging over all (water) molecules.

Finally, *vibrational* motion was quantified through the vibrational density of states $\textrm{Dos}(\omega )$ obtained from Fourier transform of the velocity auto-correlation functions,

3 $$\begin{aligned} \textrm{Dos}(\omega ) \propto \sum _{i=1}^{N} \int _{-\tau }^{\tau }\langle \underline{v}_{i}(t). \underline{v}_{i}(0) \rangle e^{i \omega t} dt\end{aligned}$$

Here, $\underline{v}_{i}(t)$ is the velocity vector of atom *i* at time *t* and $\omega$ is the vibrational frequency. The velocity autocorrelation function in (3) was calculated using the velocity trajectory of duration 50 ps, and the resulting correlation function was further Fourier transformed without using a window function.

### Vibrational analysis and spectroscopy for OH stretch modes

Equation (3) provides time-averaged information on the entire frequency distribution. Special emphasis in this work, however, is on spectroscopic signals specifically for the OH stretching region, which is sensitive to hydrogen bonding.

#### OH frequency calculations and frequency distributions

Time-dependent frequencies of local OH stretching modes were obtained using the short-window wavelet transform of the time-series analysis generated from the simulation trajectory for each system^38^. Details of the method, and applications to liquid water or to water near semiconductor surfaces are provided elsewhere^23,39–42^. The wavelet transform of a time-dependent signal *f*(*t*), unlike its Fourier transform, gives the localized frequency content over a short time interval. In the present work, as in Ref.^23^, for example, we have constructed the time-series *f*(*t*) as a complex function with its real and imaginary parts corresponding to the instantaneous fluctuation in OH bond length and the fluctuation in momentum projected along the OH bond vector at *t*, i.e.,

4 $$\begin{aligned} {f(t) = \delta r_\textrm{OH}(t) + i \delta p_\textrm{OH}(t)}\end{aligned}$$

The fluctuations are defined as $\delta q(t)=q(t)-\langle q\rangle$ where *q*(*t*) is the instantaneous property ($=r_\textrm{OH}$ or $p_\textrm{OH}$), and $\langle q \rangle$ the corresponding time average. From the time- and frequency distributions of (OH) vibrations, using a frequency binning system, also *time-averaged frequency distributions* $P(\omega )$ can be obtained as histograms for the systems of interest. The same wavelet transform (and same parameters) as in Ref.^23^ have been used to do so – see there for details. As will be shown below, the $P(\omega )$ curves obtained in this way for the OH stretching region, are similar but not identical, to the corresponding $\textrm{Dos}(\omega )$ curves obtained from Eq. (3). The OH stretch mode vibrational frequencies obtained from the wavelet-transform of the time-series has been extensively used to obtain the vibrational frequency distribution (which is an equivalent to an infrared spectrum) as well as third-order nonlinear spectrum like three-pulse photon echo peak shift and 2D-IR^39^. The mean OH stretching frequency of liquid water at 300 K from ring-polymer molecular dynamics (RPMD) was found to be 3412 $\hbox {cm}^{-1}$^40^ in comparison to average OH stretching frequency from experiments of 3407 $\hbox {cm}^{-1}$ under ambient conditions (see section Result of Ref.^43^). The timescale of decay of frequency correlation as obtained from RPMD simulations was found to be 1.12 ps as compared to the known experimentally measured timescale of 1.0 ps^44^. Similarly, temperature-dependent frequency distribution of liquid $\mathrm {D_{2}O}$ from 280-350 K obtained from wavelet transform could reproduce the high shoulder distribution at higher temperature due to increase in the population of free/dangling OD modes^37^. Even for non-centrosymmetric systems like the water-air interface, we have applied the wavelet transform to study the frequency distribution of OH modes. The OH frequency distribution of the topmost interfacial layer was found to be in close agreement with the experimentally obtained vibrational sum-frequency generation (vSFG) spectrum. The frequency distribution shows a broad peak in between 3000-3700 $\hbox {cm}^{-1}$ and a sharp peak from 3700-3900 $\hbox {cm}^{-1}$ as seen in the integrated vSFG intensity^42^.

Following earlier work^23^, we also used *time-dependent joint probability distributions* for analysis. The time-dependent joint probability distribution can be expressed as

5 $$\begin{aligned} P(\omega _{3},t_{2},\omega _{1}) = \left\langle \delta \left( \omega (t_{2})-\omega _{3} \right) \cdot \delta \left( \omega (0)-\omega _{1} \right) \right\rangle\end{aligned}$$

The joint probability distribution gives the probability that a given OH mode which was oscillating with the vibrational frequency $\omega _{1}$ evolves to a frequency $\omega _{3}$ within a time interval $t_{2}$.

#### Time-resolved 2D IR signals

Related to $P(\omega _3,t_2,\omega _1)$ are 2D IR spectra. A 2D IR spectroscopic measurement obtains the third-order polarization which is generated on irradiation of the system by three consecutive pulses at the time instants ${t_{1}}$, ${t_{2}}$ and ${t_{3}}$. Within the linear response regime we can approximate the overall third order polarization as the convolution of the response function of the system with the three pulses. Depending upon the phase of the response functions they are classified as the rephasing (${R_{rp}}$) and non-rephasing (${R_{nr}}$) response functions. In this study we will probe the vibrational dynamics of the local OH stretching modes and approximate the OH oscillators as two-level vibrational systems, with vibrational states $|0\rangle$ and $|1\rangle$, transition frequency $\omega _{10}$, and transition dipole moment $\mu _{10}$ (directed along the OH bond) connecting them. Since, the third-order response functions mentioned below are for a two-level system, the vibrational anharmonicity is not considered. Further, by making the Condon approximation, we can assume the vibrational transition dipole moment to be constant near equilibrium geometry. This allows us to reduce the four point dipole correlation function based response functions to a simpler form as mentioned below,^45,46^

6 $$\begin{aligned} R_{rp}(t_{3},t_{2},t_{1})= & \mu _{10}^{4} \ \exp {\left[ -i\langle \omega _{10}\rangle t_{3}+ i\langle \omega _{10}\rangle t_{1}\right] } \ \langle \varphi _{rp}(t_{3},t_{2},t_{1})\rangle\end{aligned}$$

7 $$\begin{aligned} R_{nr}(t_{3},t_{2},t_{1})= & \mu _{10}^{4} \ \exp {\left[ -i\langle \omega _{10}\rangle t_{3}- i\langle \omega _{10}\rangle t_{1}\right] } \ \langle \varphi _{np}(t_{3},t_{2},t_{1})\rangle\end{aligned}$$

where in actual calculations, we set $\mu _{10}=1$. Further,

8 $$\begin{aligned} \varphi _{rp}(t_{3},t_{2},t_{1})= & \exp {\left[ i\int _{0}^{t_{1}}d\tau \ \delta \omega _{10}(\tau ) -i\int _{t_{1}+t_{2}}^{t_{1}+t_{2}+t_{3}}d\tau \ \delta \omega _{10}(\tau )\right] }\end{aligned}$$

9 $$\begin{aligned} \varphi _{np}(t_{3},t_{2},t_{1})= & \exp {\left[ -i\int _{0}^{t_{1}}d\tau \ \delta \omega _{10}(\tau ) -i\int _{t_{1}+t_{2}}^{t_{1}+t_{2}+t_{3}}d\tau \ \delta \omega _{10}(\tau )\right] }\end{aligned}$$

Here, ${\delta \omega _{10}}$ is the corresponding fluctuation in the vibrational frequency of OH modes. Two-dimensional correlation spectra can be obtained by performing two-dimensional Fourier transforms of the response functions as given below,

10 $$\begin{aligned} { S(\omega _{3},t_{2},\omega _{1}) = Re\left[ S_{rp}(\omega _{3},t_{2}, \omega _{1} ) + S_{nr}(\omega _{3},t_{2}, \omega _{1} ) \right] }\end{aligned}$$

where

11 $$\begin{aligned} { S_{rp}(\omega _{1},t_{2},\omega _{3} ) = \int _{0}^{\infty }dt_{1} \int _{0}^{\infty } dt_{3}\,\exp {(i\omega _{3}t_{3}-i\omega _{1}t_{1})} \ R_{rp}(t_{1},t_{2},t_{3}) }\end{aligned}$$

and

12 $$\begin{aligned} {S_{nr}(\omega _{1},t_{2},\omega _{3} ) = \int _{0}^{\infty }dt_{1} \int _{0}^{\infty } dt_{3} \exp {(i\omega _{3}t_{3}+i\omega _{1}t_{1})} \ R_{nr}(t_{1},t_{2},t_{3}) }\end{aligned}$$

A detailed theoretical derivation of nonlinear vibrational echo spectrum as mentioned in Equation 6-12 is available in Ref.^46^. For rest of the manuscript, we refer to theoretically simulated $\mathrm {S(\omega _{3},t_{2},\omega _{1})}$ as defined in Eq. 10 which is obtained under the Condon approximation assuming OH mode to be a two-level system as 2D-IR.

## Results and discussion

### Dynamics

#### Pair distribution functions and “adsorption energies”

**Fig. 2:**
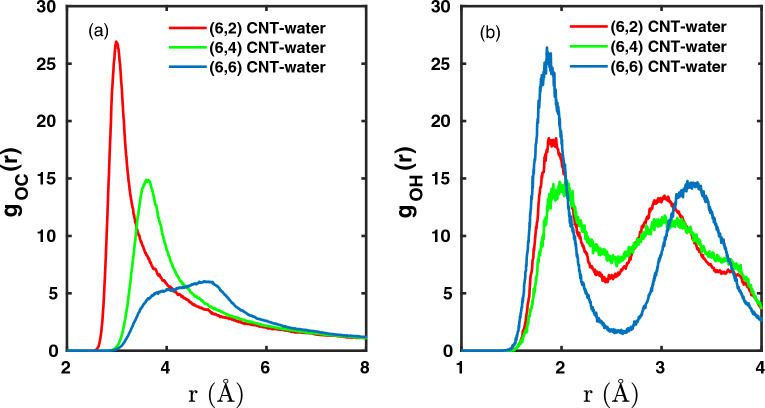
Inter-particle distribution functions for (**a**) carbon of CNTs with oxygen of water molecules, $g_\textrm{OC}(r)$ and (**b**) oxygen and hydrogen of different water molecules, $g_\textrm{OH}(r)$, obtained from trajectories in the “production phase” as described in Sec.Computational Details.

We note that although the water in our CNTs is not isotropic, we can still interpret the dynamics by inter-particle radial distribution functions of water with the CNT walls and with other water molecules. In Fig. 2(a), we show the inter-particle distribution function $g_{\textrm{OC}}(r)$ between O (of water) and C (of CNT) as a measure for the average distance *r* between the oxygen of water molecules to the carbons of CNTs.

For (6,2) and (6,4), $g_{\textrm{OC}}$ is sharply peaked at low distances, *r*, at around 3 Å (for (6,2)) and around 3.6 Å (for (6,4)), which is about half the diameter of these narrow CNTs (see Table1). In contrast for the wider (6,6) CNT, the inter-particle distribution $g_\textrm{OC}(r)$ is broad, with a larger peak around 4.8 Å and a shoulder at around 3.7 Å. We can speculate already here that the water molecules face less hydrophobic interactions with the CNT walls for (6,6) compared to (6,2) and (6,4), which may affect the strength of the H-bonds between molecules and this will have an effect on vibrational spectra in the OH region. That is indeed the case as will be shown later.

Moreover, if we examine the inter-particle distribution function $g_\textrm{OH}$ between oxygen and hydrogen of water molecules for all three cases, we see from Fig. 2(b) the first peak is centered around 1.8–2 Åindicating that any two nearest water molecules are hydrogen bound to each other. However, the minima between the first and second peak in case of (6,6) is more defined, which implies that the water molecules form anisotropic solvation network along the z-axis, such that the first and second nearest neighbor of a water molecule are unique and at specific distances. In contrast, the peaks for anisotropic solvation of water molecules in (6,2) and (6,4) are of comparable height and closer to each other. The second pair distribution peak for water molecules in (6,2) and (6,4) is centered at 3 Åas compared to 3.35 Åfor (6,6) CNT. The broadly diffused peak corresponding to second solvation shell of water molecules in case of (6,2) and (6,4) implies that the alignment of water molecules along z-axis is such that the second hydrogen of nearest water molecule and the hydrogen of second nearest water molecule make a combined contribution to the peak.

The differences in peak position, peak heights for distribution functions for (6,2), (6,4) in comparison to (6,6) indicate differences in intermolecular water-water interactions which can subsequently influence their dynamics and energetics.

Overall, the water molecules in the narrower CNTs are more squeezed than in (6,6), which can also be seen from a computed “adsorption energy” per water inside the CNT, which we define as

13 $$\begin{aligned} \mathrm { {E_{ads} = \frac{E_\text {CNT-water} - \left( E_\text {CNT} + N_\text {water} \, E_\text {water}\right) }{N_\text {water}}}}. \end{aligned}$$

Here, ${E_\mathrm {CNT-water}}$, ${E_\textrm{CNT}}$ and ${E_\textrm{water}}$ are the electronic energy of the water wire inside CNT, isolated CNT and single water molecule as obtained using the same basis set and pseudopotentials defined Sec.Computational Details by doing geometry optimization using BFGS optimizer^47^. Further, ${N_\textrm{water}}$ is the number of water molecules forming the one-dimensional wire inside the CNT. The computed ${E_\textrm{ads}}$ for the CNT of chirality (6,2), (6,4) and (6,6) were 68, -1.1 and -17.1 kJ/mol, respectively. It is evident that the water wire inside the (6,2), (6,4) are high-energy forms which support relatively faster rotational and vibrational dynamics within them, compared to (6,6), as we shall see below.

#### Translational dynamics

**Fig. 3:**
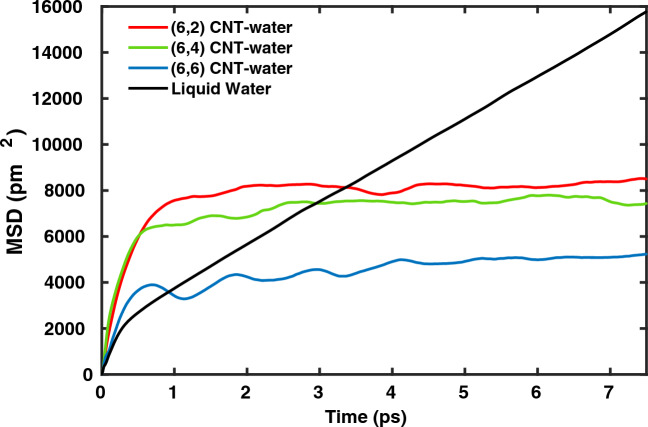
Temporal evolution of mean square displacement (MSD), along *z*, of water molecules confined within (6,2), (6,4) and (6,6) CNTs and liquid water, obtained from AIMD simulations at 300 K.

The diffusion coefficient (${D_{z}}$) of water molecules along the *z*-axis of CNTs studied in this work was calculated from Eq. (1). The mean square displacement (MSD) along the *z*-axis for the water wires within the different CNTs along with that for bulk liquid water, all at T$=300$ K, is shown in Fig. 3.

A ballistic regime at early times (up to $\sim 0.5$ ps), and a diffusive regime thereafter can be seen.

The MSDs for water in (6,2) and (6,4) CNTs are very similar. However, water molecules diffuse relatively faster in (6,6) CNT. The diffusion coefficients obtained for the CNTs with index (6,6), (6,4) and (6,2) are 1.6 $\times 10^{-10}$, 1.2 $\times 10^{-10}$ and 0.9 $\times 10^{-10} \hbox {m}^{2}$s$^{-1}$, respectively. For comparison, the diffusion coefficient of water molecules in bulk at T=300 K is 9.1 $\times 10^{-10}$ m$^{2}$s$^{-1}$ and if multiplied by a factor of 3 to take into account the three-dimensional isotropic diffusion, this is in reasonable agreement with the experimentally known value of diffusion coefficient, i.e., 23 $\times 10^{-10}$ m$^{2}$s$^{-1}$^48^. In obtaining the diffusion coefficients, we made linear fits of the four curves in Fig. 3, in the (diffusive) time interval [1,6] ps. While such linear fits are less justified for the CNTs compared to bulk water, we can confidentally say that the diffusion coefficient of the confined water is clearly reduced compared to bulk water, which seems to be a characteristic of chain-like water arrangements in thin CNTs^3,14^. The small diameter (6 to about 9 Å) and chirality of the CNTs can strongly influence the translational motion of water molecules as shown in earlier simulations^1,4,14^. The diffusion coefficient of water inside CNTs of similar diameter as obtained in Ref.^14^, for example, was reported to be in the range of 1-5 $\times 10^{-10}$ m$^{2}$s$^{-1}$ . For water molecules confined within (6,6) CNT, a relatively larger diameter causes somewhat faster translational diffusion, but is still hindered compared to the bulk.

#### Rotational dynamics

**Fig. 4:**
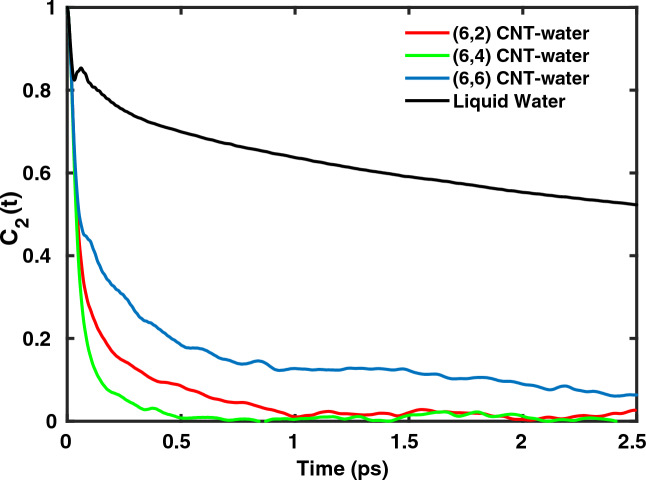
Time-dependent decay of the orientational correlation function $C_2(t)$ for $\hbox {H}_2$O dipole vectors as defined in Eq. (2), for water chains confined in (6,2), (6,4), (6,6) CNTs and liquid water, respectively.

Further we studied the rotational / reorientational dynamics of water molecules using orientational correlation functions derived from water dipole vectors as defined in Eq. (2). From Fig. 4, we note that the rotational correlation functions $C_2(t)$ of water within CNTs essentially follow a bi-exponential decay pattern. The water molecules being confined along the two lateral degrees of freedom, *x* and *y*, prefer to rapidly oscillate along the *z*-axis of the CNT. Using a bi-exponential fit^41^ to the curves (with $f(t)=C_2(t)$),

14 $$\begin{aligned} {{f(t)}= a_{0} \exp (-\frac{t}{\tau _{0}}) + (1-a_{0}) \exp (-\frac{t}{\tau _{1}})}\end{aligned}$$

gives, for ${\tau _{1}}$ of (6,2) and (6,4), (6,6), 0.70, 0.45 and 2.73 ps, respectively.

The smaller diameters of (6,2) and (6,4) imply that the confinement is more pronounced for them compared to (6,6) CNT. As a result, the water molecules in (6,2) and (6,4) exhibit faster rotational dynamics with timescale of nearly 0.5 ps. In contrast, water molecules in (6,6) undergo slower reorientational motion.

It is also evident from Fig. 3 that water molecules within the CNTs exhibit much faster rotational motion compared to bulk water predominantly owing the confinement and absence of a 3D HB network. Another difference between the reorientational dynamics of water within CNTs as compared to liquid water is the absence of initial oscillatory decay related to librational motion for the latter^49^. We note that the initial ultrafast decay up to 100 fs ($\tau _{0}$) is observable in liquid water as well as in water confined in CNTs. This decay is mainly attributed to the restricted rotational motion of water molecules with intact intermolecular hydrogen bonds. However, the hydrogen bond network of water is CNTs in under-coordinated as compared to bulk. Thus the restricted rotational motion of water molecules without breaking hydrogen bonds is seen to decay very fast in initial 100 fs and without any oscillations for water confined within CNTs.

### Vibrational analysis and spectroscopy

#### Vibrational density of states

**Fig. 5:**
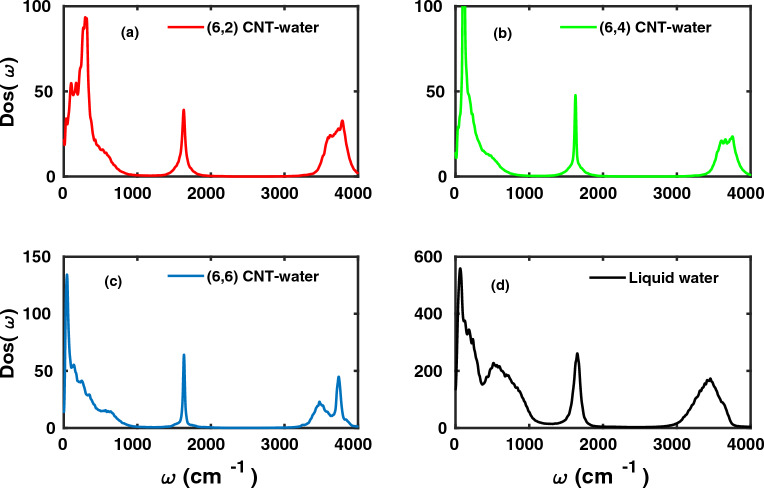
Density of states ($\mathrm {Dos(\omega )}$) of water molecules in (**a**) (6,2), (**b**) (6,4), (**c**) (6,6) CNT and (**d**) of liquid water, obtained from AIMD at 300 K.

Beyond the translational and rotational dynamics we studied in detail the vibrational dynamics of water molecules in confinement. In Fig. 5 we provide the vibrational density of states $\textrm{Dos}(\omega )$ obtained from the velocity auto-correlation functions from Eq. (3). The vibrational $\mathrm {DoS(\omega )}$ are obtained from instantaneous fluctuations in the velocity of water molecules under confinement. The forces of propagation acting on the system are computed in AIMD from DFT at each timestep which determine the coordinates and velocity of the system at next time instant. Classical force fields with predefined potentials fail to take into account the anharmonicity, intermolecular coupling and resonance effects. However, the anharmonicity of the bonds, intermolecular coupling, local solvent effects are directly incorporated in AIMD as the forces acting on the system are obtained “on-the-fly” by solving the electronic wavefunction of the system. Nevertheless, a direct comparison with the experiments may also rely on factors like experimental instrument response.

For liquid water (Fig. 5(d), one finds a broad peak situated around 3000-3700 cm$^{-1}$, which are the OH stretch vibrations. For these, we note some key differences for water in CNTs. While the liquid water peak is Gaussian-like and largely structureless, non-symmetric peaks, with one part shifted towards higher vibrational frequencies (upto 3900 cm$^{-1}$) are seen for water in CNTs. The blue-shifted portion implies water molecules which do not show extensive and strong hydrogen bonding, e.g., free / dangling OH bonds, as known from earlier work^23,50–52^. In contrast, OH modes which are hydrogen-bonded are known to oscillate at lower frequency. For the (6,6) CNT, the vibrational density of states (VDOS) shows a clear double-peak with strong peak around 3800 cm$^{-1}$ and a broad peak in the range of 3000-3800 $\mathrm {cm^{-1}}$ as a characteristic feature of interfacial water molecules^23,50–52^ with both “free” and H-bonded OH. The infrared spectroscopy of water filled single walled CNTs of diameter in range of 0.7 to 2.1 nm has also shown similar trends with a low intensity broad peak around 3400 cm$^{-1}$ corresponding to strongly hydrogen bonded water and high intensity peak around 3700 cm$^{-1}$ due to free OH modes or as described in experiments as loosely bounded water^53^.

The bending mode of water molecules in liquid water is situated around 1650 cm$^{-1}$ (Fig.5(d)). The corresponding peak is red-shifted by 25 cm$^{-1}$ under confinement within the CNTs. Further the overall FWHM (full-width at half maximum) decreases somewhat, implying gas-phase like characteristics for the bending mode. A similar red shift of 35 cm$^{-1}$ in the bending mode of water inside single-walled CNTs was experimentally found^53^.

The spectral range of 400-1000 cm$^{-1}$ is mostly attributed to the librational modes/restricted rotational motion of hydrogen-bonded water molecules^49^. We note that unlike liquid water with significant intensity and broadly spread distribution, the corresponding signals for water in (6,2), (6,4) and (6,6) CNTs have considerably lower intensity.

Finally, the spectral range of 0-400 cm$^{-1}$ is dominated by inter-molecular hydrogen bond bending and stretching motion. The peaks of water molecules in CNT show visible peak intensities mostly due to the fact that the weakly coordinated water molecule should have higher propensity to exhibit inter-molecular hydrogen bond stretching. The low energy hydrogen bonded $\mathrm {O \cdots O \cdots O}$ rotation peak situated around 40 cm$^{-1}$ is observable in all cases of confinement as in bulk. The above mentioned peak can also be seen in experimental infrared spectrum of water filled single-walled CNTs^53^

#### Closer inspection of OH stretching region: Time-averaged

We now have a closer look to the OH stretching region of confined water, first through time-averaged frequency distributions $P(\omega )$ (this chapter), then by time-resolved frequency distributions $P(\omega _3,t_2,\omega _1)$ and spectra $S(\omega _3,t_2,\omega _1)$ (in chapter Vibrational analysis and spectroscopy). In all cases, first the time-dependent vibrational frequency of the local OH stretching modes of water molecules confined within CNTs was calculated using the short-window wavelet transform of time-series analysis as described in Sec.Vibrational analysis and spectroscopy for OH stretch modes.

**Fig. 6:**
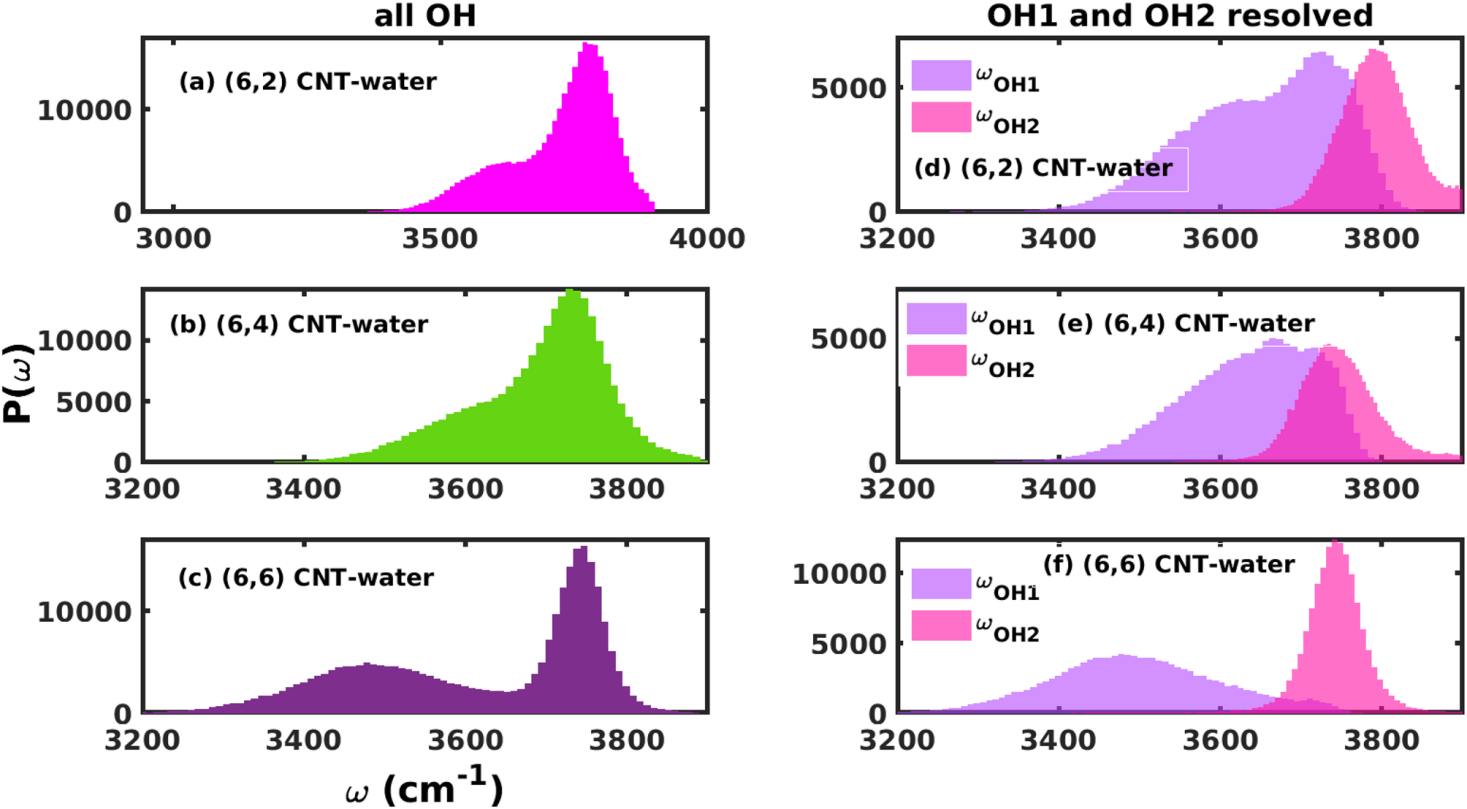
Left: Time-averaged vibrational frequency distributions $P(\omega )$ of all OH modes of water confined in CNTs of chirality (**a**) (6,2), (**b**) (6,4) and (**c**) (6,6) using wavelet transform and a binning approach (see Sec.Vibrational analysis and spectroscopy for OH stretch modes). Bins of width of 10 cm$^{-1}$ were used. Right: Frequency distributions $P(\omega _\textrm{OH1})$ and $P(\omega _\textrm{OH2})$ of stronger H-bonded (OH1) and weakly or not hydrogen-bonded (OH2) intramolecular OH modes, for the same systems.

In Fig. 6, left, we show the time-averaged distributions $P(\omega )$ of OH frequencies for water molecules for the three systems simulated. Overall, the frequency distributions $P(\omega )$ are similar to those seen in the $\textrm{Dos}(\omega )$ peaks of the OH stretch region in Fig. 5. From Fig. 6, the mean OH stretch frequencies of water molecules in (6,2), (6,4) and (6,6) CNT were found at 3720, 3690 and 3620 cm$^{-1}$, respectively. For comparison, the mean vibrational frequency of OH stretching modes in liquid water obtained from AIMD is 3422 cm$^{-1}$. There is blueshift in mean frequency of OH modes in confinement as compared to bulk water which increases as an overall effect of change in chirality index from (6,2) to (6,6) and increase in diameter of CNT from 6.21 to 8.65 Årespectively. This blueshift of nearly 200-300 cm$^{-1}$ in the vibrational frequency of OH modes of confined water molecules for all cases in comparison to bulk water is already observed in the $\mathrm {DoS (\omega )}$ in Fig.5. Further for the case of (6,6) CNT, two distinct peaks occur also in agreement with the $\mathrm {DoS (\omega )}$ shown in Fig. 5(c), while for (6,2) and (6,4) a lower-frequency feature appears as a shoulder near the higher-frequency main peak. An experimental infrared study of water filled single-walled CNTs^53^ corresponding to relative humidity of 9 $\%$ reported nearly 85 $\%$ contribution from loosely bounded water molecules to the OH stretching mode spectral region centered around 3640 cm$^{-1}$. Similarly, the frequency distribution of OH modes for one dimensional water-wire in (6,6) CNT is in very close agreement with experimentally obtained infrared spectrum corresponding to relative humidity of 30 $\%$. As water humidity increases inside CNT in experiment overall stability and strength of hydrogen bonds increases. In case of (6,6) CNT, the adsorbed water molecules are energetically more stable and thus corresponding spectrum is in agreement with infrared experiments.

The shift of frequencies towards higher values implies that the corresponding water molecules are under-coordinated compared to the bulk where water molecules form tetrahedral hydrogen bond networks^37^. While water molecules in bulk are known to form nearly four hydrogen bonds with neighboring water molecules^39^, under confinement the hydrogen-bond network is under-coordinated. By using a $\mathrm {O \cdots O}$ distance cutoff of 3.3 Å for defining the HB bonds, we found that number of HBs per water molecule in (6,2), (6,4) and (6,6) CNT to be 1.58, 1.52 and 1.80 respectively. If we revisit the time-averaged vibrational frequencies of the OH mode in the three cases it is evident that the observed distributions are a consequence of more HBs in (6,6) as compared to other two cases.

There exists asymmetry in liquid water due to the difference in the strength of hydrogen bonds formed by intra-molecular OH modes of a water molecules^54^. For water molecules confined in the CNTs, the 3D tetrahedral hydrogen bond network is disrupted, and the intra-molecular asymmetry should therefore be more pronounced and more easily observable. Out of the two intramolecular OH bonds, one, OH1 has its H atom H-bonded to a neighbor molecule along the chain, and the other one, OH2 has a H which is not or only weakly enganged in H-bonds. Both should be distinguishable by measuring the OH stretching frequencies. We thus get two time-averaged distributions $P(\omega _\textrm{OH1})$ and $P(\omega _\textrm{OH2})$ and we can define an asymmetry in OH vibrational frequencies, by a quantity $\gamma$,

15 $$\begin{aligned} \gamma = {\overline{\omega }}_\textrm{OH1} -{\overline{\omega }}_\textrm{OH2}\end{aligned}$$

where the ${\overline{\omega }}$ are mean frequencies of both types of OH bonds.

The two distributions $P(\omega _\textrm{OH1})$ and $P(\omega _\textrm{OH2})$ of water within the CNTs are shown in Fig.6, right. For water confined within (6,2), (6,4) and (6,6), the asymmetry $\gamma$ is 136, 109 and 243 cm$^{-1}$, respectively. Note that (6,6) CNT with its strong asymmetry shows well separated $P(\omega _\textrm{OH1})$ and $P(\omega _\textrm{OH2})$ distributions, which are responsible for the double-peak structure of $P(\omega )$ in Fig.6, left (c). For (6,6), the higher-frequency peak comes from the “free” OH bonds. Peaking around 3750 cm$^{-1}$, this frequency is close to the OH stretch modes of gas phase water molecules (3657 cm$^{-1}$ for symmetric and 3756 cm$^{-1}$ for antisymmetric stretch)^55^. The lower-frequency peak with maximum close to 3500 cm$^{-1}$ for (6,6), corresponds to H-bonded OH groups, with smaller force constant. It is red-shifted as mentioned relative to a free water molecule, and similar to the mean bulk water OH stretch frequency mentioned above (3412 cm$^{-1}$ according to PIMD). Note that for (6,2) and (6,4), the OH1 and OH2 peaks strongly overlap and the OH1 peaks are around 3600–3700 cm$^{-1}$, i.e., less shifted w.r.t. free water. This can be interpreted as being due to weaker H-bonds in these systems, and also the interaction with the CNT walls contributes to an overall more complicated behavior.

Pertaining to the optical activity of bonded and free/dangling OH modes as observed in the frequency distribution and power spectrum, we must emphasize that our calculations are based on the wavelet transform of time-series constructed from trajectory and Fourier transform of velocity-velocity autocorrelation function respectively. The infrared and Raman activity can be decisively ascertained by theoretically produced infrared/Raman spectrum from dipole-dipole and polarisability-polarisability autocorrelation functions. In AIMD simulations, the dipole and polarisability of the mode can be obtained by calculating the maximally localized Wannier centers (MLWCs) associated with the given mode. Being computationally expensive, the calculation of MLWCs were not performed in present work. We also note that the water molecules confined within the CNT do not have a perfect $C_{2v}$ symmetry due to frequent breaking and reformation of hydrogen bonds. Henceforth, as an qualitative argument, the selection rules for being infrared/Raman active, *i.e.* non-zero dipole moment/polarisability change are essentially satisfied in the case of dangling/free OH modes. This can also be supported by the fact that free OH modes on the water-air interface are measured in vSFG spectrum^50^

#### OH stretching region: Time-resolved frequency distributions and correlation functions

**Fig. 7:**
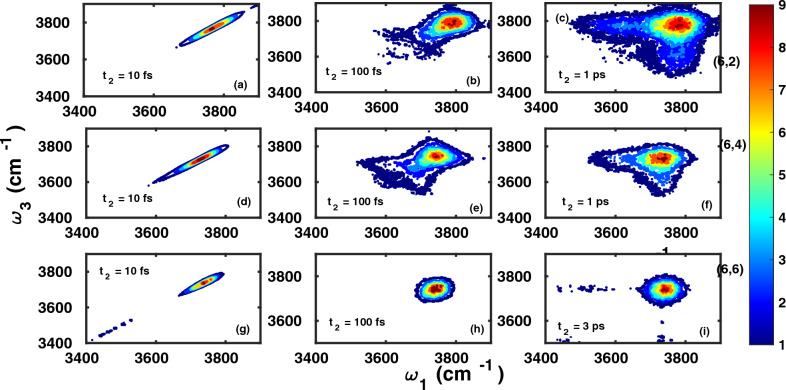
Time-resolved frequency probability distributions, $P( \omega _{3},t_{2},\omega _{1})$, Eq. (5), of OH modes confined within the CNTs of chirality (6,2) (panels **a-c**), (6,4) (**d-f**), and (6,6) (**g-i**) for the waiting times $t_{2}$ as shown in the subplots.

Further insight can be gained from the temporal evolution of OH stretching mode frequencies, which can be analyzed using the joint probability distributions $P(\omega _3,t_2,\omega _1)$ as defined in Eq. (5). Figure 7 shows this quantity for the three filled CNTs under study. We recall that $P(\omega _3,t_2,\omega _1)$ is an estimate for the probability that an OH mode oscillating at frequency $\mathrm {\omega _{1}}$, evolves after time interval ${t_{2}}$ to vibrational frequency $\mathrm {\omega _{3}}$.

From Fig. 7 we note that the OH frequencies for very small waiting times, i.e., for $t_{2}$=10 fs, are predominantly close to their initial vibrational level and thus the joint frequency distributions are essentially aligned along the diagonal axis. For longer waiting times, i.e., 100 fs, the distributions get broad indicating that OH modes are able to explore other accessible vibrational frequencies. By 1 ps for water molecules confined with the CNTs of chirality (6,2) and (6,4), the joint probability distribution is essentially spherical, indicating that spectral diffusion of vibrational states is complete for all OH modes irrespective of their initial vibrational frequency. This *spectral diffusion time* is larger for water molecules in CNT of chirality (6,6), extending up to 3 ps as closer inspection shows.

**Fig. 8:**
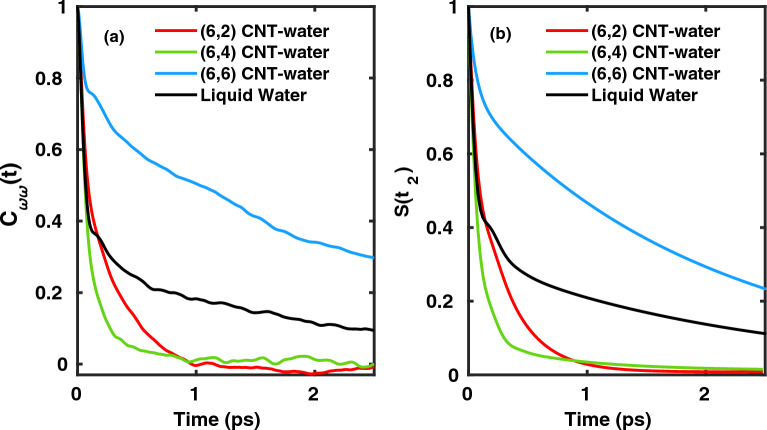
(**a**) Time-dependent decay of FTCFs (Eq. 16) of OH modes of water confined in CNTs of chirality (6,2), (6,4) and (6,6) as well as liquid water. (**b**) Same for the S3PE signal.

The different spectral diffusion times and other aspects of temporal evolution of OH modes under confinement can also, perhaps more clearly, be seen from frequency-frequency time-correlation functions (FTCF) for OH vibrations, calculated from wavelet transform, i.e.,

16 $$\begin{aligned} {C_{\omega \omega }(t) = \left\langle \delta \omega (0)\cdot \delta \omega (t) \right\rangle,} \end{aligned}$$

where ${\omega (t)}$ is the vibrational frequency of OH mode at time *t*. The FTCFs for the systems under study are shown in Fig. 8(a).

The temporal decay of correlation in the frequency of water’s OH mode in liquid water has been extensively studied and is known to be bi-phasic^39–42^, and a fit similar to Eq. (14) can be made, with $f(t)=C_{\omega \omega }(t)$ and (other) time constants $\tau _0$ and $\tau _1$. The initial ultrafast regime, related to $\tau _0$, extends up to 100 fs and is governed by librational dynamics of water molecules whereas the long tail decay, controlled by $\tau _1$, is attributed to hydrogen bond rearrangement dynamics which is also observable in Fig. 8(a), black line. For water in CNTs, the librational timescale $\tau _0$ remains similarly fast, i.e., the effect of librational motion on OH frequencies appears to be only weakly system-dependent for the models considered here. The longer timescale of correlation decay ($\mathrm {\tau _{1}}$), however, is sensitive and for (6,2), (6,4) and (6,6) CNTs we find $\tau _1=$ 0.30, 0.66 and 2.2 ps, respectively. The longer $\tau _1$ timescale for (6,6) compared to (6,2) and (6,4) parallels the longer spectral diffusion time mentioned above and is due to a slower H-bond reorganization (HB breaking / making) for (6,6) compared to (6,2) and (6,4) – which has a strong influence on OH vibrations. We suggest once more that the faster rearrangement for (6,2) and (6,4) is related to the fact that these are high-energy phases (cf. Sec.Dynamics). For comparison, the time-constant $\tau _1$ for liquid water is 2.0 ps, in agreement with earlier studies^39–42^. We have no clear explanation at the moment why $\tau _1$ for water in (6,6) is even somewhat larger than for liquid water, but this may have to do with a more stabilized 1D HB network in (6,6) compared to the more flexible 3D HB network in the liquid phase where HB bonds are frequently broken and reformed. Moreover, the number of neighboring water molecules are significantly reduced in the CNTs and thus the water wire which is stabilized in (6,6) CNT might have OH modes which prefer to stay for longer time oscillating at a particular vibrational frequency and thus slower timescale for frequency correlation loss.

#### OH stretching region: 2D IR spectra and integrated echo intensities

**Fig. 9:**
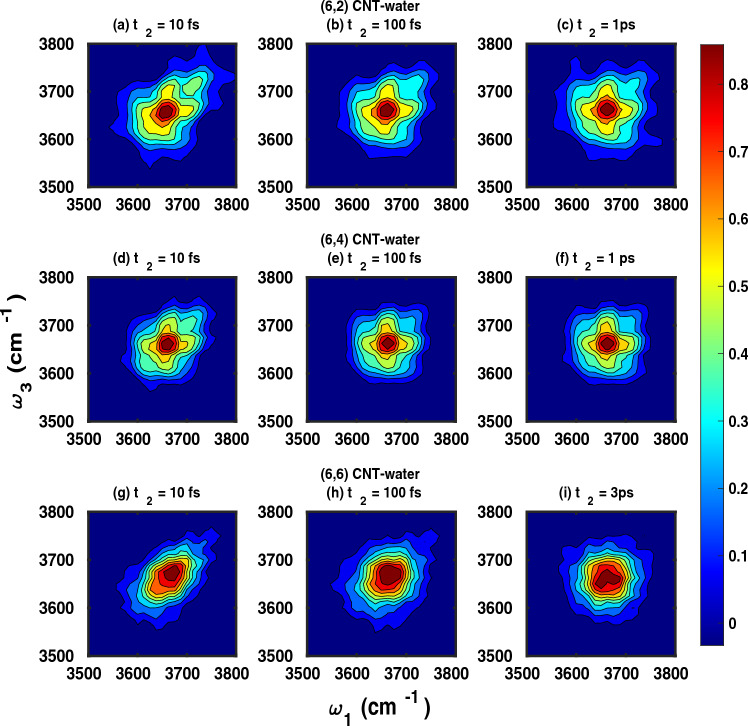
2D IR spectra ${S(\omega _{1}, t_{2}, \omega _{3})}$, Eq. (10), of OH vibrations for water molecules confined in CNTs of chirality (6,2) (top panels, (**a-c**)), (6,4) (middle panels, (**d-f**)), and (6,6) (lowest panels, (**g-i**)), at waiting times $t_2$ as indicated.

We have also studied the OH stretching vibrational dynamics of confined water using two-dimensional infrared spectra. 2D IR signals, ${S(\omega _{1}, t_{2}, \omega _{3})}$, were calculated from Eq. (10) and are shown in Fig.9, for the three CNT systems and different waiting times, $t_2$. The 2D IR spectra give similar information as the distribution functions $P(\omega _3,t_2,\omega _1)$, however, are directly related to measurable signals. A key similarity between the joint probability distribution and 2D-IR spectra is that both provide timescale of spectral diffusion in OH stretching mode spectrum which is governed by hydrogen-bond rearrangement dynamics. In principle, the broadening of IR spectrum due to inhomogenous broadening is captured by both metrics and thus the 2D-IR and joint probability distributions looks similar for the time interval longer than timescale of inhomogenous broadening ( here solvent/hydrogen bond network rearrangement). The homogeneous broadening of the infrared spectrum due to natural lifetime effects and rotational effects are not captured in joint probability distributions. Accordingly, for timescales at which the lineshapes are strongly dependent upon homogeneous broadening, 2D-IR and joint probability distributions are not comparable. This can be seen in our results for case of 10 fs. Here the probability distributions are diagonally elongated but do not have any characteristic features along the anti-diagonal. The anti-diagonal broadening of lineshape as seen in 2D-IR for 10 fs is a consequence of homogeneous broadening. To summarize, the broadening of 2D-IR spectrum is governed by both broadening mechanisms while joint probability distributions capture only inhomogenous broadening.

In Fig. 9(a)-(c), the 2D IR spectra of water molecules confined within (6,2) for the waiting times ${t_{2}}=$ 10, 200 and 1000 fs are shown. At low waiting times like 10 or 200 fs, the spectrum is predominantly aligned along the diagonal showing that the final vibrational levels are strongly correlated to initial states. However, for a waiting time of 1 ps, the 2D IR spectrum is quasi-spherical indicating that all the vibrational states are accessible within this time interval and the correlation memory of the initial vibrational state is totally lost. Similar statements can be made for water in (6,4) based on the 2D IR spectra shown in Fig. 9(d)-(f). According to Fig. 9(g)-(i), the 2D IR spectra of the local OH stretch modes of water in (6,6) shows relatively slower vibrational spectral diffusion as the 2D IR spectra is still aligned along the diagonal for a waiting time of 1 ps. At $t_2=$ 3 ps, we observe that the spectrum became spherical. The somewhat longer spectral diffusion time for (6,6) compared to (6,2) and (6,4), is consistent with the picture developed in the last section, Sec.Vibrational analysis and spectroscopy.

Admittedly, the longer spectral diffusion time of (6,6) is not so easy to see from Fig. 9, but again a more quantitative picture can be obtained from another quantity, namely the slope of three pulse photon echo intensity in this case^39,40^, which is also available from the integrated echo intensity in experiments. The integrated echo intensity is defined as

17 $$\begin{aligned} {I(t_{1},t_{2}) = \int _{0}^{\infty }dt_{3}\left| R_{rp}(t_{1},t_{2},t_{3}) \right| ^2}\end{aligned}$$

The short-time slope of the integrated echo intensity at initial time $t_{1}=0$ (S3PE)^39,40^, can be written as

18 $$\begin{aligned} {S(t_{2})= \frac{\partial I(t_{1},t_{2}) }{\partial t_{1} }\bigg |_{t_{1}=0}} \quad . \end{aligned}$$

It is easily observable from Fig. 8(b), where the S3PE is shown for the three systems considered here, that this signal quite precisely resembles the time-correlation functions ${C_{\omega \omega }(t)}$ in Fig. 8(a). We again use the bi-exponential fit function (14) to obtain the long timescale (${\tau _{1}}$) of loss in spectroscopic observable, i.e., the S3PE of water molecules confined with CNTs, which are 0.32, 0.98 and 2.2 ps (6,2), (6,4) and (6,6) CNT, respectively. The timescale associated with S3PE of liquid water is 2.1 ps, as also reported in earlier studies^37^. The timescales in Fig. 8(b) are in fact quite similar to those found from decay of $C_{\omega \omega }(t)$, and the same rationalization as before is appropriate. The OH vibrational frequency and the associated time-resolved spectra like S3PE are strongly governed by rearrangement of the local hydrogen bond network. This is also evident from time-averaged DoS, frequency distributions as well as time-resolved 2D-IR spectra. We can also ascertain it by using continuous hydrogen bond lifetime correlation function to get hydrogen-bond lifetime for confined water insise CNTs as shown in Fig S2 of supporting information^23^. The HB lifetime for water confined within CNT of chirality (6,2), (6,4), (6,6) and bulk water were found to be 0.30, 0.20, 1.12 and 1.02 ps respectively. We note that the hydrogen bond between the water molecules confined in (6,6) CNT are relatively long-lived compared to bulk water as well as water wires within CNT of chirality (6,2) and (6,4) as also seen from FTCF and S3PE calculations. A pair of water molecules were inferred to be hydrogen bonded, if the interparticle $\mathrm { O\cdots O}$ distance was less than 3.3 Å.

In this study, we have explored the dynamics of one-dimensional water-wire confined within CNTs of different curvature(chirality) and diameters. The resultant water dynamics is a consequence of the diameter and electronic interactions based on curvature (or chirality) in between the CNT and water-wires. While ab-initio MD of water confined in neutral featureless cylinder is not feasible, the effect of curvature can be deciphered by doing simulation of water confined in two-dimensional graphene slab. We will address this issue of the role of chirality and curvature by doing simulation of water confined in CNTs, two- dimensional graphene and graphene analogues like $\mathrm {C_{2}N}$, $\mathrm {C_{3}N_{4}}$ in our future works. We also note several experiments have reported graphene to be mildly hydrophilic in nature^56^. However, curvature, diameter, single/double wall of carbon nanotube, interaction of $\pi$ electrons of carbon atoms with the water molecules and van der Waals interactions make carbon nanotubes overall different in comparison to a graphene surface. This is particularly relevant in determining if CNTs like graphene are hydrophobic or otherwise. The addition of dopants and modified chemical structure of graphene is also an important factor in this regard. In a future AIMD based study we aim to address this issue by investigating the local structure, translational, rotational and vibrational dynamics of water confined with graphene slabs and its two-dimensional doped equivalents mentioned above and compare them with water inside CNTs.

## Summary and conclusions

To summarize, we have studied by DFT-based AIMD the translational, rotational and vibrational dynamics of water molecules, confined in chain-like geometries in narrow CNTs of different chirality, i.e., (6,2), (6,4) and (6,6). The water molecules show slower translational diffusion along the CNT axis as compared to bulk water but can undergo fast reorientational relaxation in the absence of strong three-dimensional hydrogen bond networks. Rather, the water chains form quasi-1D hydrogen bond networks, exhibiting two different types of OH bonds within individual molecules: dominantly H-bonded or dominantly “free”. Further, the vibrational dynamics of water molecules with CNT shows a strong dependence on the chirality and diameter of the CNTs. The overall vibrational frequency distribution of local OH stretch shows a blue-shift of 200-300 cm$^{-1}$ under confinement as compared to bulk liquid water. However, in particular for (6,6), two distinct OH stretching regions are seen, corresponding to H-bonded and “free” OH bonds. Moreover, we find that for the narrower (6,2) and (6,4) CNTs, in which water is spatially more constrained laterally and interacts more strongly with the hydrophobic walls, water molecules show ultrafast OH vibrational decay with a timescale of less than 1 ps whereas for water molecules within (6,6) CNT, the OH frequency correlation loss extends up to 3 ps. 2D IR spectra and signals related to them, like the short-time slope of the integrated echo intensity, appear as powerful tools to unravel details of vibrational dynamics of water in confinement.

On a more speculative side, the observed differences in the vibrational dynamics of water for not too wide CNTs with different chirality should make it feasible to tailor these carbon-based materials for different industrial applications like water-splitting or desalination of water depending upon overall stabilization within the CNTs.

## Supplementary Information

Supplementary Information.

## Data Availability

The data generated are available from the corresponding author upon reasonable request.

## References

[1] Srivastava, A., Abedrabbo, S., Hassan, J. & Homouz, D. Dynamics of confined water inside carbon nanotubes based on studying tetrahedral order parameters. *Sci Rep* **14**, 15480 (2024). 38969700 10.1038/s41598-024-66317-1 PMC11226439

[2] Chatzichristos, A. & Hassan, J. Current understanding of water properties inside carbon nanotubes. *Nanomaterials* **12**, 174 (2022). 35010123 10.3390/nano12010174 PMC8746445

[3] Chakraborty, S., Kumar, H., Dasgupta, C. & Maiti, P. B. Confined water: structure, dynamics, and thermodynamics. *Acc. Chem. Res.* **50**, 2139–2146 (2017). 28809537 10.1021/acs.accounts.6b00617

[4] Hummer, G., Rasaiah, J. C. & Noworyta, J. P. Water conduction through the hydrophobic channel of a carbon nanotube. *Nature* **414**, 188–190 (2001). 11700553 10.1038/35102535

[5] Kalra, A., Garde, S. & Hummer, G. Osmotic water transport through carbon nanotube membranes. *Proc. Nat. Acad. Sci.* **100**, 10175–10180 (2003). 12878724 10.1073/pnas.1633354100 PMC193535

[6] Kohler, M. H. & Bordin, J. R. Surface, density, and temperature effects on the water diffusion and structure inside narrow nanotubes. *J. Phys. Chem. C* **122**, 6684–6690 (2018).

[7] Maniwa, Y. et al. Water-filled single-wall carbon nanotubes as molecular nanovalves. *Nature Mater* **6**, 135–141 (2007). 17237788 10.1038/nmat1823

[8] Gelb, L. D., Gubbins, K. E., Radhakrishnan, R. & Sliwinska-Bartkowiak, M. Phase separation in confined systems. *Rep. Prog. Phys.* **62**, 1573–1659 (1999).

[9] Bonthuis, D. J. et al., Theory and simulations of water flow through carbon nanotubes: prospects and pitfalls. *J. Phys.: Condens. Matter* **23**, 184110 (2011). 10.1088/0953-8984/23/18/184110 21508478

[10] Uematsu, Y., Netz, R. R., Bocquet, L. & Bonthuis, D. J. Crossover of the power-law exponent for carbon nanotube conductivity as a function of salinity. *J. Phys. Chem. B* **122**, 2992–2997 (2018). 29489370 10.1021/acs.jpcb.8b01975

[11] Gordillo, M. & Marti, J. Hydrogen bond structure of liquid water confined in nanotubes. *Chem. Phys. Lett.* **329**, 341–345 (2000).

[12] Marañón Di Leo, J., and Marañón, J., Confined water in nanotube. *J. Mol. Struct. THEOCHEM* **623 **, 159—166 (2003). 3019 –13022

[13] Falk, K. et al. Molecular origin of fast water transport in carbon nanotube membranes: superlubricity versus curvature dependent friction. *Nano Lett.* **10**, 4067–4073 (2010). 20845964 10.1021/nl1021046

[14] Gokura, L. et al. The peculiar size and temperature dependence of water diffusion in carbon nanotubes studied with 2D NMR diffusion-relaxation D- spectroscopy. *Biomicrofluidics* **14**, 034114 (2020). 32595817 10.1063/5.0005398 PMC7305942

[15] Hassan, J. et al. Ultrafast stratified diffusion of water inside carbon nanotubes; direct experimental evidence with 2D D-T2 NMR spectroscopy. *J. Phys. Chem. C* **122**, 10600–10606 (2018).

[16] Nanok, T. et al. Structure and dynamics of water confined in single-wall nanotubes. *J. Phys. Chem. A* **113**, 2103–2108 (2009). 19115825 10.1021/jp8088676

[17] Loche, P. et al. Giant axial dielectric response in water-filled nanotubes and effective electrostatic ion-ion interactions from a tensorial dielectric model. *J. Phys. Chem. B* **123**, 10850–10857 (2019). 31765168 10.1021/acs.jpcb.9b09269

[18] Liu, X., Pan, X., Zhang, S., Han, X. & Bao, X. Diffusion of Water Inside Carbon Nanotubes Studied by Pulsed Field Gradient NMR Spectroscopy. *Langmuir* **30**, 8036–8045 (2014). 24951088 10.1021/la500913r

[19] Briganti, G. et al. Neutron scattering observation of quasi-free rotations of water confined in carbon nanotubes. *Sci Rep* **7**, 45021 (2017). 28327621 10.1038/srep45021 PMC5361194

[20] Cametti, C., De Luca, F. & Parmentier, A. Radiowave dielectric investigation of water confined in channels of carbon nanotubes. *J. Chem. Phys.* **137**, 094908 (2012). 22957595 10.1063/1.4749571

[21] Byl, O. et al. Unusual hydrogen bonding in water-filled carbon nanotubes. *J. Am. Chem. Soc.* **128**, 12090–12097 (2006). 16967958 10.1021/ja057856u

[22] Weinwurm, M. & Dellago, C. Vibrational spectroscopy of water in narrow nanopores. *J. Phys. Chem. B* **115**, 5268–5277 (2011). 21280603 10.1021/jp109037q

[23] Ojha, D., Penschke, C. & Saalfrank, P. Vibrational dynamics and spectroscopy of water at porous g- surfaces. *Phys. Chem. Chem. Phys.* **26**, 11084–11093 (2024). 38530253 10.1039/d3cp05964b

[24] Su., W. et al., Chirality-dependent electrical transport properties of carbon nanotubes obtained by experimental measurement. *Nat Commun* **14**, 1672 (2023). 10.1038/s41467-023-37443-7 PMC10039901 36966164

[25] Matsuda, Y., Tahir-Kheli, J. & Goddard, W. A. Definitive band gaps for single-wall carbon nanotubes. *J. Phys. Chem. Lett.* **1**, 2946–2950 (2010).

[26] Rajter, R. F. et al. Chirality-dependent properties of carbon nanotubes: electronic structure, optical dispersion properties. *Hamaker coefficients and van der Waals-London dispersion interactions RSC Adv.* **3**, 823–842 (2013).

[27] Kühne, T. D. et al. CP2K: An electronic structure and molecular dynamics software package - Quickstep: efficient and accurate electronic structure calculations. *J. Chem. Phys.* **152**, 194103 (2020). 33687235 10.1063/5.0007045

[28] Bussi, G., Donadio, D. & Parrinello, M. Canonical sampling through velocity rescaling. *J. Chem. Phys.* **126**, 014101 (2007). 17212484 10.1063/1.2408420

[29] VandeVondele, J. et al. Quickstep: fast and accurate density functional calculations using a mixed Gaussian and plane waves approach. *Comput. Phys. Commun.* **167**, 103–128 (2005).

[30] VandeVondele, J. & Hutter, J. Gaussian basis sets for accurate calculations on molecular systems in gas and condensed phases. *J. Chem. Phys.* **127**, 114105 (2007). 17887826 10.1063/1.2770708

[31] Goedecker, S., Teter, M. & Hutter, J. Separable dual-space Gaussian pseudopotentials. *J. Phys. Rev. B* **54**, 1703–1710 (1996). 10.1103/physrevb.54.1703 9986014

[32] Krack, K. Pseudopotentials for H to Kr optimized for gradient-corrected exchange-correlation functionals. *Theor Chem Acc* **114**, 145–152 (2005).

[33] Becke, A. D. Density-functional exchange-energy approximation with correct asymptotic behavior. *Phys. Rev. A* **38**, 3098–3100 (1988). 10.1103/physreva.38.3098 9900728

[34] Lee, C. T., Yang, W. T. & Parr, R. G. Development of the Colle-Salvetti correlation-energy formula into a functional of the electron density. *Phys. Rev. B* **37**, 785–789 (1988). 10.1103/physrevb.37.785 9944570

[35] Grimme, S., Antony, J., Ehrlich, S. & Krieg, H. A consistent and accurate ab initio parametrization of density functional dispersion correction (DFT-D) for the 94 elements H-Pu. *J. Chem. Phys.* **132**, 154104 (2010). 20423165 10.1063/1.3382344

[36] Pestana, L. R., Mardirossian, N., Head-Gordon, M. & Head-Gordon, T. Ab initio molecular dynamics simulations of liquid water using high quality meta-GGA functionals. *Chem. Sci.* **8**, 3554–3565 (2017). 30155200 10.1039/c6sc04711d PMC6092720

[37] Ojha, D. & Chandra, A. Temperature dependence of the ultrafast vibrational echo spectroscopy of OD modes in liquid water from first principles simulations. *Phys. Chem. Chem. Phys.* **21**, 6485–6498 (2019). 30840019 10.1039/c8cp07121g

[38] A. Semparithi, A. & Keshavamurthy, S. Intramolecular vibrational energy redistribution in DCO (): Classical-quantum correspondence, dynamical assignments of highly excited states, and phase space transport. * Phys. Chem. Chem. Phys.* **5**, 5051—5062 (2003).

[39] Ojha, D., Karhan, K. & Kühne, T. D. *On the hydrogen bond strength and vibrational spectroscopy of liquid water Sci Rep* **8**, 16888 (2018). 30443040 10.1038/s41598-018-35357-9 PMC6237855

[40] Ojha, D., Henao, A. & Kühne, T. D. Nuclear quantum effects on the vibrational dynamics of liquid water. *J. Chem. Phys.* **148**, 102328 (2018). 29544291 10.1063/1.5005500

[41] Ojha, D. & Kühne, T. D. Vibrational dynamics of liquid water in an external electric field. *Phys. Chem. Chem. Phys.* **25**, 13442–13451 (2023). 37092187 10.1039/d3cp01128c

[42] Ojha, D., Henao, A., Zysk, F. & Kühne, T. D. Nuclear quantum effects on the vibrational dynamics of the water-air interface. *J Chem. Phys.* **160**, 204114 (2024). 38804494 10.1063/5.0204071

[43] S. A. Corcelli, S. A. & Skinner, J. L. Infrared and Raman Line Shapes of Dilute HOD in Liquid H2O and D2O from 10 to 90 C * J. Phys. Chem. A* **109**, 6154—6165 (2005). 10.1021/jp0506540 16833955

[44] Eaves, J. D., Loparo, J. J., , Fecko, C. J., Roberts, S. T., Tokmakoff. A. & P. L. Geissler, P. L. Hydrogen bonds in liquid water are brokenonly fleetingly. *Proc. Nat. Acad. Sci.* **102**, 13019—13022 (2005). 10.1073/pnas.0505125102 PMC1201598 16135564

[45] Mukamel, S., Principles of nonlinear optical spectroscopy. (Oxford–New York, 1995).

[46] Schmidt, J. R., Corcelli, S. A. & Skinner, J. L. Pronounced non-Condon effects in the ultrafast infrared spectroscopy of water. *J. Chem. Phys.* **123**, 044513 (2005). 16095375 10.1063/1.1961472

[47] Byrd, R. H., Lu, P. H., Nocedal, J. & Zhu, C. Y. A limited memory algorithm for bound constrained optimization. *SIAM J. Sci. Comput.* **16**, 1190–1208 (1995).

[48] Harris, K. R. & Woolf, L. A. Pressure and temperature dependence of the self diffusion coefficient of water and oxygen-18 water. *J. Chem. Soc., Faraday Trans. 1.* **76**, 377—385 (1980).

[49] Heyden, M. et al. Dissecting the THz spectrum of liquid water from first principles via correlations in time and space. *Proc. Nat. Acad. Sci.* **107**, 12068–12073 (2010). 20566886 10.1073/pnas.0914885107 PMC2901429

[50] Ojha, D. & Kühne, T. D. “On-the-fly’’ calculation of the vibrational sum-frequency generation spectrum at the air-water interface. *Moleucles* **25**, 3939 (2020). 10.3390/molecules25173939 PMC7504776 32872259

[51] Ojha, D. & Kühne, T. D. Hydrogen bond dynamics of interfacial water molecules revealed from two-dimensional vibrational sum-frequency generation spectroscopy. *Sci Rep* **11**, 2456 (2021). 33510246 10.1038/s41598-021-81635-4 PMC7844302

[52] Ojha, D., Kaliannan, N. K. & Kühne, T. D. Time-dependent vibrational sum-frequency generation spectroscopy of the air-water interface. *Commun Chem* **2**, 116 (2019).

[53] Bernardina S. D. et al. Water in carbon nanotubes: the peculiar hydrogen bond network revealed by infrared spectroscopy *J. Amer. Chem. Soc.* **138**, 10437—10443(2016). 10.1021/jacs.6b02635 27455124

[54] Kühne, T. D. & Khaliullin, R. Z. Electronic signature of the instantaneous asymmetry in the first coordination shell of liquid water. *Nat Commun* **4**, 1450 (2013). 23385594 10.1038/ncomms2459

[55] Tennyson, J. et al. Experimental energy levels of the water molecule. *J. Phys. Chem. Ref. Data* **30**, 735–831 (2001).

[56] Kozbial, A., Zhou, F., Li, Z., Liu, H. & Li, L. Are graphitic surfaces hydrophobic?. *Acc. Chem. Res.* **49**, 2765–2773 (2016). 27935273 10.1021/acs.accounts.6b00447

