# Supplementary material for: Dynamics and vibrational spectroscopy of quasi-one dimensional water wires inside carbon nanotubes of different diameter and chirality
Source: Sci Rep. 2025 Aug 1;15:28144. doi: 10.1038/s41598-025-14266-8 (PMC12317096; doi:10.1038/s41598-025-14266-8)
Supplement: Supplementary file 1 — Supplementary Information. [file 41598_2025_14266_MOESM1_ESM.zip › si_zip/SI.pdf]

Supporting Information : Dynamics and vibrational spectroscopy of quasi-one dimensional water wires inside carbon nanotubes of different diameter and chirality

Deepak Ojha and Peter Saalfrank

Institute of Chemistry, Potsdam University, Karl-Liebknecht-Strasse 24-25, D-14476 Potsdam-Golm, Germany

*Correspondence should be addressed to: ojha.deepak@uni-potsdam.de

S1 Integrated Radial Distribution Function

The local structure and ordering of water confined within the CNTs can also be analyzed using integrated radial distribution functions which is given mathematically as,

$$\mathrm{G_{OH}(r)} = \int_0^{\mathrm{r}} 4\pi\rho\grave{\mathrm{r}}^2 \mathrm{g}(\grave{\mathrm{r}})\mathrm{d}\grave{\mathrm{r}}, \tag{S1}$$

where $\mathrm{G_{OH}(r)}$ is integrated radial distribution function, ρ is the density of simulation box and $\grave{r}$ is the distance from the central atom. It is evident in Fig.**??** that the $\mathrm{G_{OH}(r)}$ is more oscillatory and evolves at faster rate for (6,6) CNT which indicates higher structural ordering within the one-dimensional wire. Further, the value of $\mathrm{G_{OH}(r)}$ at 4 Å for (6,2), (6,4) and (6,6) CNTs is 3.7, 3.6 and 4.0 respectively. This also implies that the local water structure is more anisotropic in case of (6,2) and (6,4) as compared to (6,6) CNT. Further, the overall local density of water is lower in case of (6,2) and (6,4) as compared to (6,6) CNT. For the given value of $\mathrm{G_{OH}(r)}$, it also implies nearly two water molecules can be found around a water molcule within the distance of 4 Å.

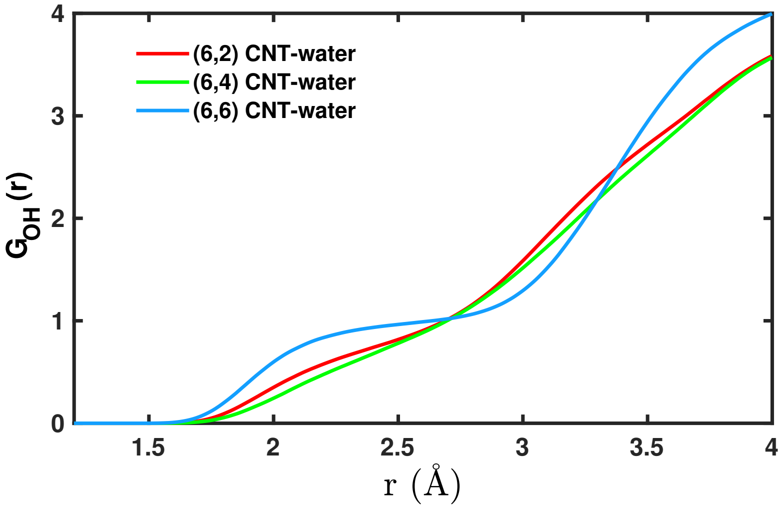

Figure S1: Integrated radial distribution functions as obtained using Equation 13 from the O $\cdots$ H radial distribution function which are duly shown in Figure 2 of the main article.

S2 Hydrogen Bond Correlation Function

The HB continuous lifetime correlation function S_{HB}, which denotes the probability that an initially H-bonded pair of water molecules remains continuously intact until time t, can be defined mathematically as,

$$S_{HB}(t) = \frac{\langle h(0)H(t)\rangle}{\langle h(0)^2\rangle} \quad . \tag{S2}$$

Here, $H(t)$ and $h(t)$ are hydrogen populations labels and for a pair of hydrogen-bonded water molecules can be defined as, $h(t)$ takes a value of 1 if they are hydrogen bonded at time instant t. Further, if the two water molecules are continuously hydrogen-bonded for a time-interval say from time instant t= 0 to t, $H(t)$ is 1. Time-dependent decay of S_{HB} for water molecules in CNTs of different chirality and bulk is shown in Fig.**??**. We use the bi-exponential fit function as shown in Eq. 15 of main article to find the hydrogen bond lifetime.

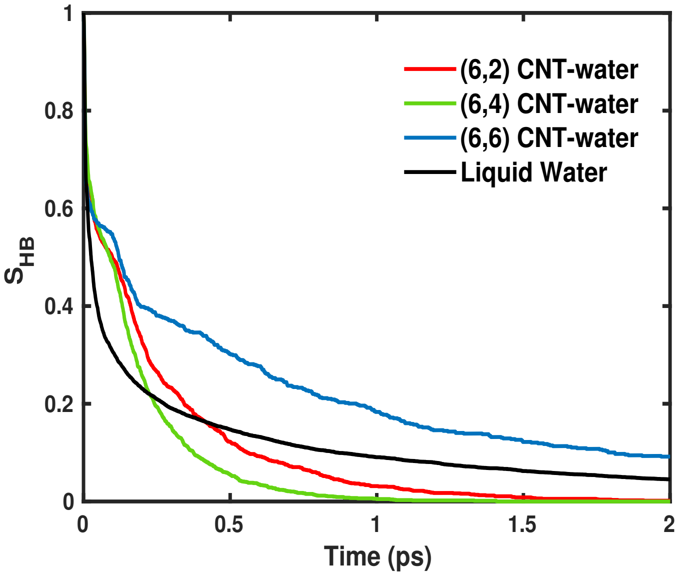

Figure S2: Continuous hydrogen bond lifetime correlation functions S_{HB} of water molecules confined in CNTs of chirality (6,2) , (6,4), (6,6) and in bulk.
