# Supplementary figures and images for: Dynamics and vibrational spectroscopy of quasi-one dimensional water wires inside carbon nanotubes of different diameter and chirality
Source: Sci Rep. 2025 Aug 1;15:28144. doi: 10.1038/s41598-025-14266-8 (PMC12317096; doi:10.1038/s41598-025-14266-8)

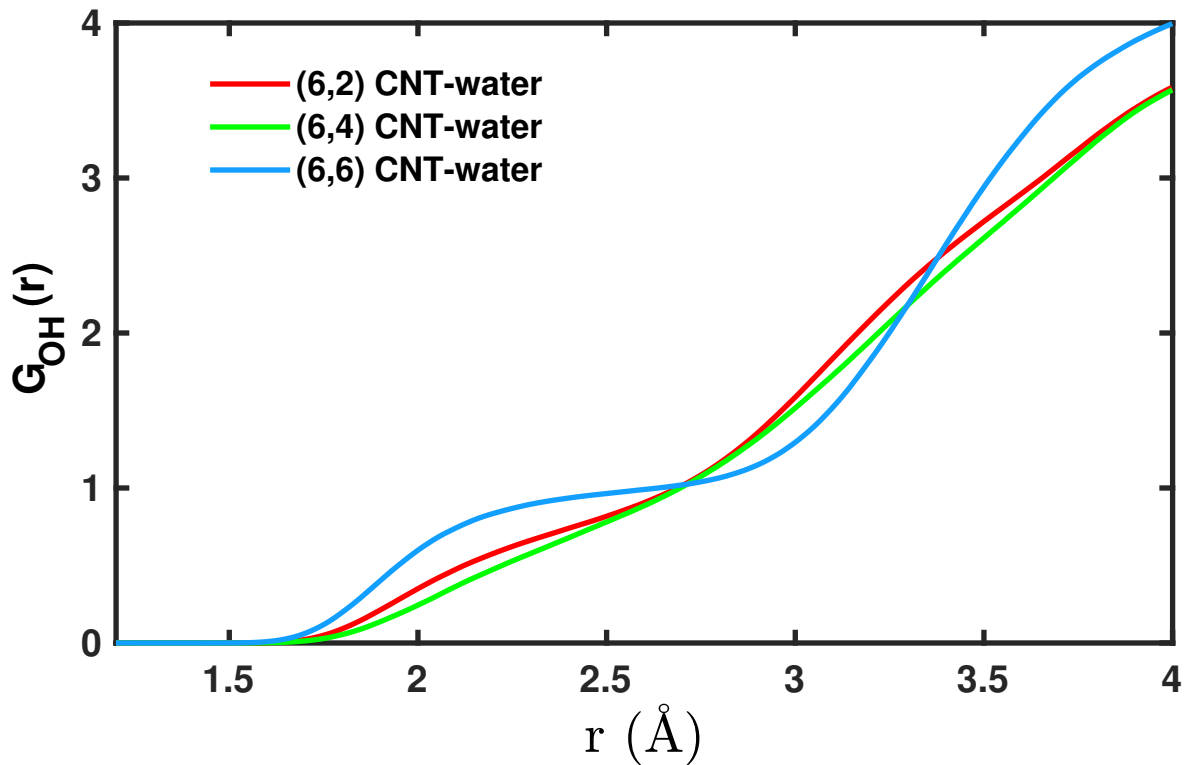

Supplement: Supplementary file 1 — Supplementary Information. [file 41598_2025_14266_MOESM1_ESM.zip › si_zip/SI1-eps-converted-to.pdf]

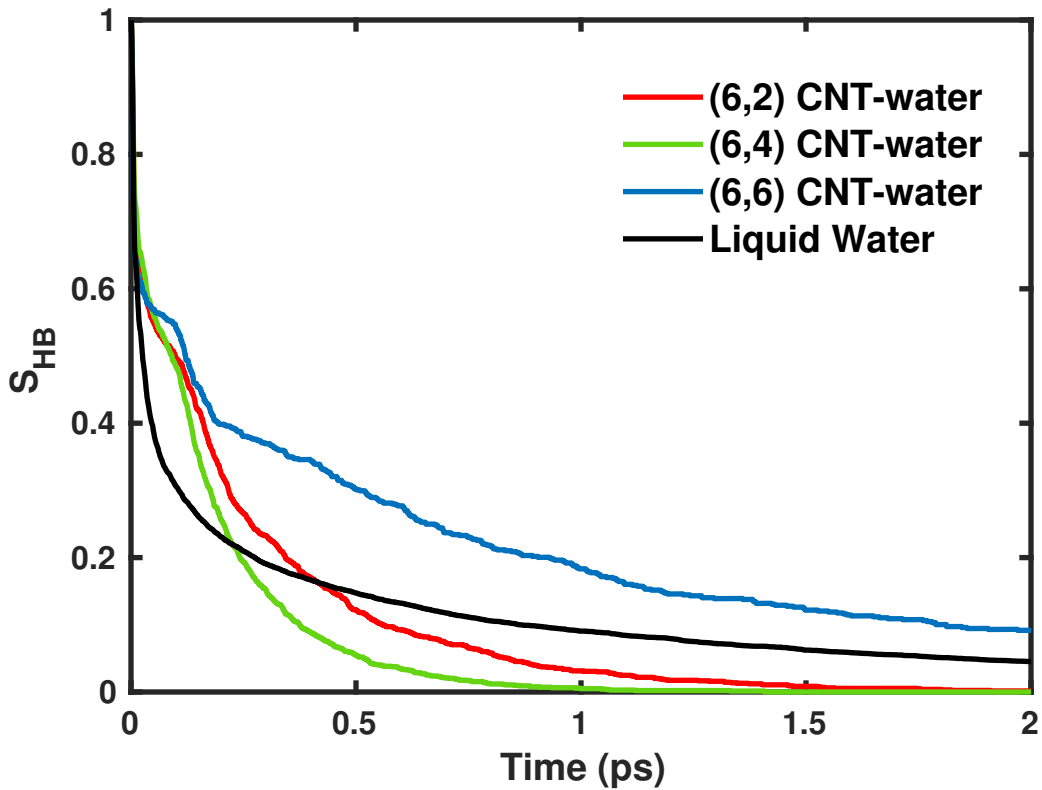

Supplement: Supplementary file 1 — Supplementary Information. [file 41598_2025_14266_MOESM1_ESM.zip › si_zip/SI2-eps-converted-to.pdf]
